# Unravelling Conformational Aspects of Milk Protein Structure—Contributions from Nuclear Magnetic Resonance Studies

**DOI:** 10.3390/foods9081128

**Published:** 2020-08-16

**Authors:** Tatijana Markoska, Todor Vasiljevic, Thom Huppertz

**Affiliations:** 1 Advanced Food Systems Research Unit, Institute for Sustainable Industries and Liveable Cities, College of Health and Biomedicine, Victoria University, Melbourne VIC 8001, Australia; tatijana.markoska@live.vu.edu.au (T.M.); todor.vasiljevic@vu.edu.au (T.V.); 2 FrieslandCampina, 3818 LE Amersfoort, The Netherlands; 3 Food Quality and Design Group, Wageningen University and Research, 6808 WG Wageningen, The Netherlands

**Keywords:** NMR, milk protein, protein structure, casein, whey protein

## Abstract

Changes in the molecular structure and association of milk proteins lead to many desirable (under controlled conditions) or undesirable characteristics of dairy products. Several methods have been used to study the structure of milk proteins and changes therein in different environments. Whey proteins are an excellent model for secondary structure studies using circular dichroism (CD), Fourier-transform infrared spectroscopy (FTIR) and tertiary structure studies using X-ray crystallography and nuclear magnetic resonance (NMR). However, caseins, the most abundant protein class in milk, are far more difficult to characterize. The tertiary structure of caseins cannot be observed by X-ray crystallography due to the inability to crystallize caseins. However, NMR is an appropriate approach for structural elucidation. Thus far, NMR was applied on specific peptides of individual caseins of the molecules including phosphoserine centers and colloidal calcium phosphate. The literature focuses on these parts of the molecule due to its importance in building the sub-unit particles involving individual caseins and calcium phosphate nanoclusters. This review focuses on present structural studies of milk proteins using NMR and their importance in dairy processing.

## 1. Introduction

Milk is a biological fluid secreted by the mammary gland with the primary function to complete the nutritional requirements of neonates. In the dairy industry, milk is processed for maintaining safety and extending the shelf-life but is also further processed for production of different dairy products and ingredients. Milk proteins play an essential part in obtaining high quality products when appropriate processes are applied. The structures of milk proteins often undergo transformations during manufacturing processes, which may influence the quality of the final product [1]. The distribution of the amino acids in the polypeptide chain in the protein molecule is of great importance to their conformational arrangements and consequent functionality during dairy processing.

The proteins in milk are classified in two main groups, i.e., the whey proteins and the caseins and these groups are likely the most studied food proteins to date. The major whey proteins in milk are α-lactalbumin (α-LA) and β-lactoglobulin (β-LG) and considering their nutritional importance, molecular size (relatively small globular proteins) and heat sensitivity, they have been frequently studied for structural elucidation and changes therein [1,2,3,4,5]. The caseins occur mainly in the form of casein micelles and their complex structure has been intriguing dairy scientists for decades. The structure of the micelles consists of four casein types, i.e., α_s1_-, α_s2_- and β-casein located primarily in the interior and κ-casein located on the exterior of the micelles. The primary role of κ-casein is the stabilization of the micelles against aggregation. The internal structure is stabilized by calcium phosphate clusters, which bind the phosphoserine regions of caseins and thus maintain the internal structure of the micelle [6]. During processing, these internal and external interactions may be altered, leading to structural modifications of individual caseins and casein micelle structure. 

**Figure 1:**
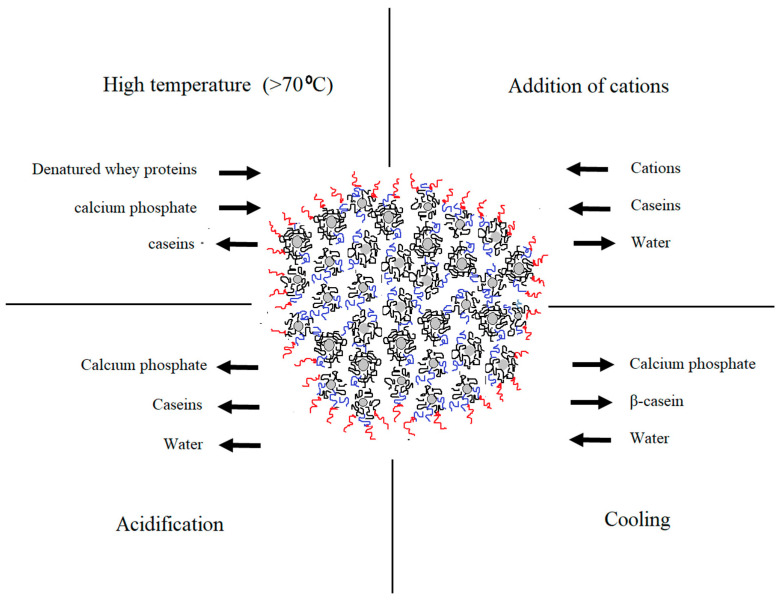
Changes in the native equilibrium of casein micelle in particular physio-chemical conditions during dairy processing (adapted from Gaucheron [11]).

Understanding the complexity of casein arrangement in the micelle is essential for the process control. Even though the substructure of the micelle was extensively studied [7,8,9,10], some aspects of the internal and external organization, especially its changes during processing, remain unclear. Changes in the micellar equilibrium take place during processing steps including addition of acids, heat treatment, cooling, pressuring, renneting, addition of cations etc. [1]. Certain physio-chemical changes that take place during some of the dominant technological processes are illustrated in Figure 1. Modified conditions affect the micelle in a different manner and intensity. Understanding these changes is important for achieving consistent product quality and process control. 

The functional properties of milk proteins are dependent on their structural organizations. Thus, any changes in the protein structure induced by a modified environment in the milk systems affect their properties. As previously discussed, casein micelle has a complex structure where each individual casein has an important function to maintain its stability. The functional properties of casein micelle are closely related to its surface and interior properties [1]. Hence, the C-terminal of κ-casein, including the casein macropeptide (CMP), comprising of residues 106–169, is located on the exterior of the micelle. This region contains a negative charge that arises from phosphorylation and glycosylation which provide steric stabilization and thus prevent the close approach of other micelles [6]. In addition, the amino acids of κ-casein that are responsible for disulphide interactions (Cys_11_ and Cys_88_) are located close to the surface of the micelle. However, this equilibrium can be disrupted when different processing conditions are applied which result in structural reorganization of milk proteins. In addition, the macropeptide is not so dense and thus when the milk equilibrium is disrupted, individual proteins can enter or exit the micelle using the micellar water channels [9]. Some of these modifications are a dissociation of β-casein from the micelle during cooling [12], causing the liberalization of CMP as a result of bond cleavage (105–106) by chymosin [13] and penetration of denatured β-LG inside the κ-casein layer and formation of covalent bonds with free Cys residues during milk pre-warming [14]. The interior of the micelle also undergoes structural reorganizations that affect protein functionality, including post-coagulation changes during cheese making [1] or dissociation of caseins and insolubilization of calcium phosphate at high temperatures [14]. All these modifications are the result of protein unfolding, exposing of their active sides and involvement of different interactions which may lead to aggregation and gelation. The structural changes of milk proteins lead to important outcomes in the processing performance of the final milk product. 

To elucidate milk protein structure and changes therein, the most frequently applied techniques include circular dichroism (CD) and Fourier-transform infrared spectroscopy (FTIR), which focus on various elements of the secondary structure [15]. CD records the signal arising from the peptide bond (170–240 nm) and aromatic residues (260–320 nm) of the proteins. Being a non-destructive and suitable for aqueous solutions, CD has been successfully applied on structural studies on milk proteins [16,17,18]. However, the method is more reliable for observation of helical structures than β-sheets, and therefore more applicable for globular proteins such as β-LG and α-LA [19]. In addition, the applicability of CD is also limited by the fact that only a limited number of buffers can be used in sample preparation. Caseins have open and unordered conformation and this can bring difficulties in the application of CD [20]. 

FTIR has been successfully applied in secondary structure studies of milk proteins using an ATR (attenuated total reflectance) cell depicting C=O and N-H stretching of the molecules [20,21,22]. However, FTIR only provides information of the presence of elements of the secondary structure in the observed system without clear differentiation of which part of the molecule is responsible for it. X-ray crystallography uses the three-dimensional density pattern of the molecule in forming an X-ray pattern and can record the atomic distance with accuracy of 0.1–0.2 Å using resolution of 1.5–2 Å range. However, this method requires the protein molecule to be crystallized. Hence, globular proteins, including α-LA and β-LG, are valid structural models for X-ray crystallography and their three-dimensional structure was successfully established at 1.8 Å resolution using this technique [23]. Caseins, due to their high surface hydrophobicity, heterogenicity and structural flexibility cannot be crystallized and thus are not suitable for structural studies by X-ray crystallography [24].

Methods such as FTIR can show the changes in the proteins as a result of processing or altered environments, but a more sophisticated approach, such as NMR, is needed to evaluate the reasons of the structural rearrangements of individual proteins and the possibility of their control. However, the complexity in theoretical and technical manipulation, including the high instrumental cost, often brings a hesitation to its selection. In comparison to X-ray crystallography, NMR can provide structural information almost under any condition applicable with a minimal sample preparation. Hence, this makes NMR an important technique for studying casein conformation. 

Current knowledge describes only a few NMR structural studies on individual caseins, specific peptide fragments to be exact, concentrating only on their phosphoserine regions due to their importance in interactions with colloidal calcium phosphate and creation of a micellar skeleton [16,25,26,27,28,29,30,31,32,33,34]. Caseins possess a rather random structure that takes flexible conformations and thus are difficult models for structural studies. On the other hand, NMR has been shown to be a reliable technique to ascertain structural properties and modifications of many proteins. NMR provides information of the position of individual atoms which can present the spatial orientation of the molecule. This technique was proven to be reliable for structural studies on whey proteins, providing detailed information of the position of individual protons and, hence, the amino acid location in the polypeptide chain [35]. At present, the protein data bank (PDB) contains 10,520 resolved structures using the NMR method out of the 165,957 reported solved conformations of proteins (rcsb.org).

Many studies have been reported in the literature that include structural elucidation of milk proteins using NMR, including predominately conformational structures of whey proteins and only some studies on caseins structure. The focus of this review is to elaborate on the importance of understanding conformational changes of milk proteins during processing and consequently their impact on the quality of the products, and how these changes can be ascertained with emphasis on suitability and application of NMR as a cutting-edge approach in elucidating protein conformations.

## 2. NMR Approach for Structural Elucidation of Proteins

Proteins are composed of amino acids organized in a polypeptide chain, which can be in a folded or flexible organization in order to function properly. The tertiary structure of proteins has become relatively easy to predict based on a known amino acid sequence and complete spatial organization, which can be established by multidimensional NMR experiments. Consequently, high resolution NMR spectroscopy can be used to observe structural characteristics of many proteins, including milk proteins. The most studied nuclei are ^1^H, ^15^N and ^31^P. Each nucleus gives a specific chemical shift in the spectral view. For a complex molecular structure such as that of a protein, one-dimensional NMR is not sufficient for identification of structural features; therefore, higher dimensions are applied. The interpretation of these higher-order spectra can be challenging and dependent on the molecular weight of the protein and the method used. NMR studies on proteins with molecular weight up to 20 kDa had become a routine due to improvements in technology. This refers to a greater availability of experiments in higher dimensions (three- and four-dimensional methods), improvement in the radiofrequency, field strength, isotopic labelling, different NMR probes and software [36].

For NMR studies of large molecules (20–50 kDa), including proteins, molecular/isotopic labelling techniques are available. The development of the NMR spectroscopy produced new techniques for optimal labelling of proteins. Many approaches for isotopic labelling have been reported, which makes the selection of an appropriate method difficult. Tugarinov et al. [37] proposed four approaches for successful labelling of proteins, i.e.,:

(1) For a backbone assignment, including ^13^C_β_ nuclei, the best approach is full labelling of ^15^N, ^2^H and ^13^C samples obtained from D_2_O based growths. The measurements are performed on protein dispersed in H_2_O after ^2^H > ^1^H exchange;
(2) For Leu, Ile δ_1_ and Val methyl groups and measurements of ^3^J_CγCO_ scalar coupling and nuclear Overhauser effect (NOE) connectivity (NH-CH_3_; HN-HN distance), the most appropriate labelling procedure is considered to be linearized ^13^C spin system including ((U-^15^N,^2^H,^13^C), Leu, Val (^13^CH_3_, ^12^CD_3_), Ileδ_1_(^13^CH_3_));
(3) A methyl labelling scheme similar to step 2, but including different carbon positions ^12^C ((U-^15^N,^2^H), Leu, Val (^13^CH_3_, ^12^CD_3_), Ileδ_1_ (^13^CH_3_)), should be used for measurements of ^3^J_CγN_ coupling and NOE connectivity (CH_3_-CH_3_);
(4) Methyl labelling as ^13^CHD_2_- labelled proteins for detecting methyl ^13^C relaxation rates.

In order to achieve a complete structural view of the observed protein by NMR, several steps need to be followed. These include chemical shift assignment, nuclear Overhauser effect (NOE) coupling and relaxation measurements for internal mobility of the proteins [38]. The first step of structural identification presents an individual chemical shift of the observed nuclei. For milk proteins, the most observed nuclei are ^1^H, ^15^N and ^31^P, which produce characteristic chemical shifts, measured in ppm. Moreover, when the chemical shift assignment is available, appropriate combinations of NMR experiments can produce information of the structure and dynamics of the observed protein. 

**Figure 2:**
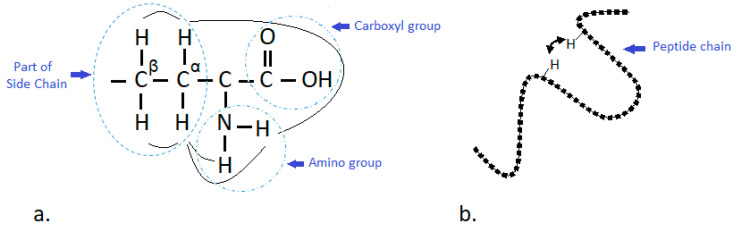
Through bound (scalar) coupling (**a**); through space coupling—nuclear Overhauser effect (NOE) (**b**).

For sequential connectivity among the nuclei in proteins, some of the most used experiments are ^1^H-^15^N and ^1^H-^13^C heteronuclear single quantum correlation (HSQC), ^1^H-^1^H correlation spectroscopy (COSY) and ^1^H-^1^H total correlation spectroscopy (TOCSY) experiments [39]. The ^1^H-^15^N HSQC and ^1^H-^13^C HSQC methods record only one signal for every amino acid or the backbone N-H and C-H chemical shift, respectively [40]. This excludes the Pro residues, due to their lack of amide proton in the molecule. The COSY spectra give information of the position of α-protons in the molecule [41]. TOCSY has an important role for localization of amino acids in the spectra predominately in the amino/fingerprint region where every amino acid appears in a specific pattern [42]. These methods give information of the neighboring atom that interacts through various bonds (Figure 2a). However, this approach only provides information about which nuclei are connected through bonds and is not sufficient for structural studies. The best performance is obtained when all experiments are recorded in H_2_O/D_2_O solution in order to reduce the rapid exchangeability of the amide protons with the solvent [43].

The second step and most important part of the structure elucidation is a NOE experiment. The spectra from nuclear Overhauser effect spectroscopy (NOESY) are obtained through space coupling between protons which are located in the spatial distance less than 5 Å [35] (Figure 2b). NOE spectra were used for the first time for sequence specific resonance assignments for proteins by Wüthrich [35] providing two types of NOE distance among protons including a strong NOE with an upper distance limit of ≤2.5 Å and a weak NOE distance of ≤4 Å. The quantitative distance measurements of the NOE experiments depend on proton/proton distance (*r*) and rotation correlation time (*τ_c_*) (Equation (1)) [38].

(1) $$\mathrm{NOE}\;\propto \;\frac{1}{\left(r^{6}\right)}\times f\left(\tau_{c}\right)$$

**Figure 3:**
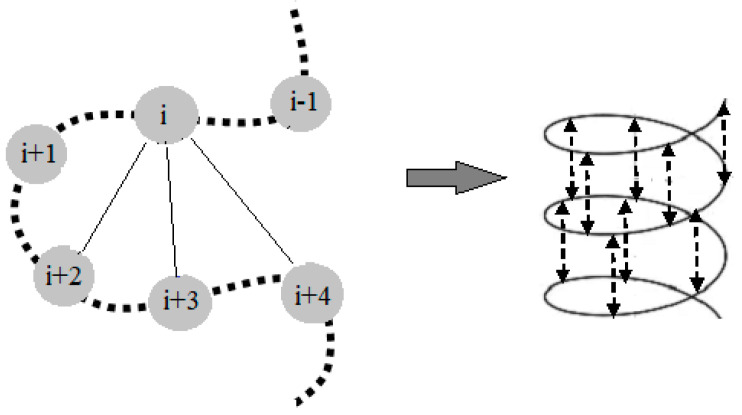
Regular NOE patterns for secondary stricture determination of α-helix.

Wüthrich et al. [43] proposed three groups of distance constraints obtained from the NOESY spectra. The first group includes characterization of the secondary structure of the protein by closely spaced nuclei or backbone H_α_ and H_β_ atoms. The second group includes distance constraints in the sequence between H_α_ and H_β_ nuclei from further apart residues in the polypeptide chain which is important for observation of extended polypeptide structures and β-sheets or α-helix. The final group includes the distance constraints among the hydrogen atoms on the side chains which characterize the tertiary structure of the molecule. The connectivity between neighboring amino acids is established by *d_AB_*(*i,j*) presenting the distance between hydrogen atoms (A and B) located in position i and j, respectively. Depending on the spectral region observed, the NOE can provide information of distance between protons in amide region *d_NN_*(*i,j*), α-protons and amide *d_αN_*(*i,j*), β-protons and amide *d_βN_*(*i,j*), α-α-protons *d_αα_(i.j)* and α-β-protons *d_αβ_*(*i,j*) [44]. Moreover, the distance between protons located at the nearby amino acid in the sequence is presented as a sequential distance, e.g., *d_αN_*(*i*,*i*+1; *i*+2, *i*+3…) [44]. The distance constraints present between the backbone and the H^β^ of the residues, which are closely positioned in the polypeptide chain, can give information of the specific secondary structure [43]. Moreover, the distance between the backbone and the H^β^ residues that are further apart in the polypeptide chain can give information of the existence of super secondary structures, i.e., β-sheet or α-helix. The cross peaks in the NOESY spectra give information of the distance between two observed protons in the peptides and thus provide information of the existence of possible structures. A simple presentation of the through space connectivity in an α- helix is presented in Figure 3. 

NOE experiments also give information of ϕ and ψ dihedral angle conformations via spin–spin coupling constraints or J-coupling constraints by the Karplus relationship [45]. The ϕ and ψ dihedral angles arise from different combinations of steric effects that took place between the residues of the same amino acid and between the side chains from different amino acids in the polypeptide chains. These angles also result from aroused hydrogen bonds during secondary structure interactions [46]. This information is an essential parameter for backbone conformation determination, including α-helices and β-sheets. 

The final step or relaxation measurements observe the recovery time for a population of spins in the observed nucleus after turning off the induced radiofrequency. In dairy products, NMR relaxation studies are mainly used to observe the water mobility and water holding capacity of the products. This includes studies that observe the state of free water and water attached to the protein matrix in the dairy products. In addition, for hydrogen as the most abundant nuclei in the organic components, two observing parameters are being obtained including spin–lattice relaxation time with a time constant T_1_ and spin–spin relaxation time with a time constant T_2_. Proton relaxation studies were explained in detail by Mariette [47] giving an overview of different applications and examples of NMR relaxation studies, diffusion coefficient measurements and magnetic resonance imaging (MRI) on dairy products. The relaxation measurement studies observe the water distribution and dynamics in the milk products, mainly with regard to the interactions among proteins and water in milk.

## 3. NMR Studies on Structure of Milk Proteins

### 3.1. Whey Proteins

When it comes to NMR studies on milk proteins, whey proteins have been studied more extensively than caseins. The main focus has been on understanding their thermal instability with α-LA and β-LG, as the predominant whey proteins, being the most unstable. As globular proteins, α-LA and β-LG have served as absolute structural models for NMR studies. Complete structural assignments have been reported in literature using predominately 2D, 3D NMR and isotopic labelling. Structural characterization and the NMR methods used are further discussed in this review. 

#### 3.1.1. α-Lactalbumin

α-LA exists as two domains including A domain, organized in four helixes and 3_10_-helix, and B domain consisted of triple anti-parallel β-sheets and 3_10_-helix [48]. α-LA was observed to exist in a molten globule state during the transition process under denaturing conditions in the early stages of refolding [49]. The NMR spectra of the molten globule of α-LA give broad linewidths and poor chemical shifts [50,51,52]. The broad linewidths of the NMR spectrum correspond to a compact and folded structure of the molecule [50] and well-defined sharp peaks correspond to unfolded parts of the protein [51,53]. Predominant NMR methods for studying the molten globule structure are N-H HSQC [51,54] and NOE connectivity for 2,3 D methods [52,53,55]. 

The structure of α-LA was studied by Alexandrescu et al. [52,56]. They used 2D NMR methods in combination with photochemically-induced dynamic nuclear polarization (PCIDNP) to observe the aromatic rings and cluster formation in amino acids in the molecule. The sequence-specific assignments among protons with aromatic rings were detected based on NOE connectivity [56]. In their next work they applied 2D NMR spectroscopy to observe the acid-denatured molten globule structure of α-LA (101–110) by using the sequential NOE connectivity for structural assignment and observed nonspecific random coil structure of the molten globule state of α-LA [52]. The unfolding transition of the molten globule of α-LA was observed in higher dimension by Schulman et al. [51] using ^15^N-labelled human α-LA, as well as 2D and 3D-edited NMR methods. This study confirms the unfolding transition of the molten globule of α-La in the presence of a denaturant, resulting in a non-cooperative collapsing of the polypeptide chain. 

Later, Forge et al. [3] used NMR in combination with CD and mass spectroscopy to observe a refolding of α-LA after chemical denaturation. They observed that the addition of Ca^2+^ after denaturation promotes conversion of the protein from a partially folded state to the native state, which is in line with studies reported by Berliner et al. [57] showing the importance of cation-interactions for α-LA. The study was performed using fully labelled α-LA protein for real-time NMR experiments (kinetics measurements) and 3D NMR methods (HSQC-TOCSY and HSQC-NOESY) for sequential assignment. Complete sequential assignments of the chemical shift were performed, and then deposited in the BioMagRes data bank (code-4332). They identified the presence of two state processes of association for refolding from the denatured to native state. Moreover, α-LA has served as a model for NMR studies, the structural studies for sequential assignments of this whey protein that are listed in Table 1.

#### 3.1.2. β-Lactoglobulin

Bovine β-LG, due to its availability and great abundance, presents a remarkable model for molecular studies [2,70,71,72,73] and has been the most extensively studied milk protein for structural modelling using NMR. At pH below 3, the naturally existing dimeric form of β-LG dissociates into a monomeric form, which can retain the native conformation even at a pH value as low as 2.26 [71]. The first NMR resonance assignment of β-LG was performed by Molinari et al. [17], who revealed a highly structured β-sheet core for the monomeric form of β-LG at pH 2. At this pH, the monomer is unfolded, forming a β-sheet and random coils observed from numerous overlapping peaks. A similar study at pH 2 was published by Ragona et al. [18], who detected a β-core and 11 hydrophobic residues located around Trp_19_ and facing towards the interior of the monomer. Both studies used 1D and 2D NMR experiments (DQF-COSY, TOCSY and NOESY) without any isotopic labelling. Uhrínová et al. [63] for the first time proposed a completely resolved structure of a recombinant form of variant A of β-LG using a solution state of NMR. The study was performed using ^13^C, ^15^N-labelled proteins and different 3D heteronuclear pulse selective NMR techniques. The findings reported eight antiparallel β-sheets organized in a barrel and surrounded by α-helix. This study served as basic knowledge for further studies, which involved characterization of the structure and dynamics of β-LG. The coordinates of this study are available under code 1DV9 in PDB.

Another important study that observed the structure and kinetics of the folding of β-LG was published by Kuwata et al. [61], who also studied the monomeric state of the protein at pH 2. Their findings regarding the monomeric structure of β-LG were similar to those by Uhrínová et al. [63] with 8 antiparallel barrel-oriented β-sheets and one major α-helix; however, Kuwata et al. [61] also pointed out a Tanford transition of β-LG confirming rigid behavior of three β-sheets located under the α-helix and prompt fluctuation of the N and C terminals of the molecule. Kuwata et al. [61] performed their study using ^13^C,^15^N labelled β-LG and heteronuclear 2D and 3D NMR experiments. Structure calculation was completed using 1143 distance restraints for φ angle based on HNHA, χ_1_ angle based on HNHB data and hydrogen bonds based on NOE data and solvent exchange [61]. The coordinates from this study are available at PDB under the code Icj5. The Tanford transition of β-LG, which results from the pH variation, was later studied in detail using heteronuclear NMR spectroscopy by Sakurai et al. [64]. Complete analysis of the NMR studies related to structural changes of β-LG are listed in Table 1. 

### 3.2. Caseins

Contrary to major whey proteins, caseins as the main group of milk proteins have been less studied by NMR. Several studies observed the secondary structure orientation of defined regions of the polypeptide chains of individual caseins. This refers to the phosphorylated regions of the molecule of multi-phosphorylated motif Ser(P)-Ser(P)-Ser(P)-Glu-Glu, which has been shown to be critical for the interaction with amorphous calcium phosphate [74]. The peptide sequence with the Ser(P) residues that are of great importance in stabilization of casein phosphate complexes are the following [29]:

αs_1_-CN (59-79): -Gln^59^-Met-Glu-Ala-Glu-Ser(P)-Ile-Ser(P)-Ser(P)-Ser(P)-Glu-Glu-Ile-Val-Pro-Asn-Ser(P)-Val-Glu-Gln-Lys^79^-

β-CN(1-25): -Arg^1^-Glu-Leu-Glu-Glu-Leu-Asn-Val-Pro-Gly-Glu-Ile-Val-Glu-Ser(P)-Leu-Ser(P)-Ser(P)-Ser(P)-Glu-Glu-Ser-Ile-Thr-Arg^25^-

αs_2_-CN(1-21): -Lys^1^-Asn-Thr-Met-Glu-His-Val-Ser(P)-Ser(P)-Ser(P)-Glu-Glu-Ser-Ile-Ile-Ser(P)-Gln-Glu-Thr-Tyr-Lys^21^-

αs_2_-CN(46-70): -Asn^46^-Ala-Asn-Glu-Glu-Glu-Tyr-Ser-Ile-Gly-Ser(P)-Ser(P)-Ser(P)-Glu-Glu-Ser(P)-Ala-Glu-Val-Ala-Thr-Glu-Glu-Val-Lys^70^-

These sequences have been studied extensively using the NMR for secondary structure identification, binding preferences for cations (Ca^2+^) and aggregation preferences [25,26,27,28,29,30,31,32,33,75,76]. Most of the studies were focused on amino acids sequence analysis of αs_1_-CN and β-CN, as the predominant caseins in milk and are thus important building blocks of the casein micelle. Complete assignment of the individual caseins using NMR methods has not been published so far. The following section summarizes the main studies that involve solution NMR for structural assignments of the four caseins (αs_1_-, αs_2_-, β- and κ-casein). Some of the studies involving structural elucidation using NMR are listed in the Table 2. The current structural studies performed using NMR of individual casein fragments are discussed in the following part of this review. 

#### 3.2.1. β-Casein

β-CN is a calcium-sensitive protein and binding of calcium ions to phosphorylated residues (five SerP residues) lead to its precipitation [6]. Β-CN was predicted to have no or little secondary structure including α-helix 7–20%, 15–33% β-sheet, 20–30% β-turns and 20–25% polyproline II structure [20,78,79,80]. β-CN is strongly amphipathic with hydrophilic N-terminus (1–40), moderate hydrophobicity in the middle section (41–135) and highly hydrophobic C-terminus (136–209) [6]. 

The N-terminus of the polypeptide chain of β-CN has been of great interest to NMR studies due to phosphoserine motif (Ser_15_, Ser_17_, Ser_18_, Ser_19_, Glu_20,_ Glu_21_) located in this part of the molecule, which is an important center of phosphorylation. The N terminus of the β-CN (β-CN f(1–25) was studied using NMR by Tsuda et al. [25] and Wahlgren et al. [26,75], who all used a purified peptide obtained after tryptic hydrolysis of β-CN. The NMR experiments were performed in H_2_O/D_2_O (90:10) and presence of calcium [25,26,75]. The main NMR methods used for these studies were 2D, including COSY, TOCSY and NOESY with adjusted phase and pulse sequence and water suppression. However, neither of these studies had observed existence of a specific structure on the observed peptide. In addition, Tsuda et al. [25] did not present any evidence for existence of β-turns in the peptide. From the NOE sequential assignments performed by Wahlgren et al. [26,75] it has been established that the N-terminus of the β-CN has multiple conformations with great flexibility. 

Cross et al. [27] reported structural features of the peptide β-CN f(1–25) in the presence of cations, whereas a detailed structural model of β-CN f(1–25) complexed with amorphous calcium phosphate (ACP) was reported by Cross et al. [29]. Cross et al. [27] focused on the complete sequence specific assignment of the peptide in the presence of ammonium, sodium and calcium ions and of the dephosphorylated peptide with sodium ions. All the samples were prepared using 10 mM of peptide and specific preparation protocol depending of the ions and method used [27]. The existence of a cation-dependent specific structure was observed in sequential and non-sequential NOE connectivity in the amide (fingerprint), and the Hα region of the NOESY/ROESY spectra. Moreover, four structures were identified in β-CN peptide (1–25) in the presence of calcium, including one loop structure (Arg_1_-Gly_4_) and three β-turns (Val_8_-Glu_11_, SerP_17_-Glu_20_ and Glu_21_-Thr_24_) [27]. 

The aforementioned findings by Cross et al. [27] were extended by Cross et al. [29] in further conformational studies of the same peptide (β-CN f(1–25)) complexed with ACP, giving the importance of calcium phosphate in the formation of the nanoclusters in the casein micelle. For the purpose casein phosphopeptide-ACP complexes were prepared as described by Reynolds [81] with a peptide concentration of 1.0–4.5 mM. NMR experiments included 2D methods (DQF-COSY, TOCSY and NOESY) using the States–Habercorn method [82] and the standard sequential assignment protocol [35,83]. The sequential assignment was completed in the fingerprint region of the NOESY spectra (NOEs and ^3^J_HNHα_ coupling constant) with mixing time of 250 ms where all residues of the complex were assigned. The proposed molecular model of CPP-ACP complex predicted an existence of a “patch” on the surface of the model consisting of Pro_9_, Ile_12_, Val_13_ and Glu_5_ [29]. Moreover, it was proposed that the entire length of the peptide is involved in interactions with ACP due to many negatively charged residues. Hence, the Ser(P) motif does not appear to be the sole calcium binding motif for the complexation with calcium phosphate [29]. These findings using NMR experiments were a great contribution to the basic knowledge of the structural organization of the β-CN molecule. However, as previously discussed, the main focus to date has been on the N-terminus of the molecule and the sequential assignment of the whole molecule of β-CN has not been reported to date. 

#### 3.2.2. α_s1_-Casein

α_s1_-CN is also a calcium-sensitive casein with eight phosphorylated residues and a secondary structure consisting of, on average, 5–20% α-helix structure, 17–46% β–sheet and 29–35% β-turn structures [32,76,84]. This casein presents approximately 40% of total casein in bovine milk and is of great importance due to its association with calcium phosphate during the formation of the casein micelle. NMR was applied as a structural method, predominately for observations on parts of the N-terminus, i.e., αs1-CN f(1–23) and f(59–79) [27,28,30,31,32] and the C-terminus, i.e., α_s1_-CN f(136–196), as a region with strong affinity for self-association in the presence of salts [77]. 

α_s1_-CN f(1–23) was studied by Malin et al. [32] and selected as an important part of the αs_1_-CN molecule due to the high tendency for self-association [6]. In their study, Malin et al. [32] used NMR in combination with FTIR, CD and molecular modelling. The performed NMR experiments were homonuclear shift correlations including NOESY and TOCSY with mixing times 300 and 124 ms, respectively. For the heteronuclear experiments, HSQC was used for observing correlations among ^1^H and ^13^C nuclei. TOCSY spectra were used for observing amino acid patterns in the fingerprint region (NH-Nα), and HSQC was used for detection of cross peaks in the alpha region (Cα-Hα) and for identification of the Pro peak position. NOESY was used for sequential short-range NOE connectivity in the fingerprint region for all the residues except prolines. The Pro residues were observed to be in trans position that may lead to lower conformational fluctuations. The main observed structure was the poly-L-proline II (PPII) structure which has an important role in casein–casein interactions [32]. 

Another fragment of α_s1_-CN that was used for structural studies using NMR was the phosphoserine region α_s1_-CN f(59–79). Huq et al. [30] published complete sequence assignments of αs_1_-CN fragment (59–79) in the presence of five moles of calcium per mol peptide and pH values of 4.06, 6.08 and 7.45. The NMR methods used were DQF-COSY, TOCSY and NOESY using the States–time-proportional phase incrementation (TPPI) method [85] for a phase sensitive mode. A standard procedure was used for a resonance assignment [35,83]. The intra-residue cross-peaks in the fingerprint region of the NOESY spectra were recorded in the best sequential assignments for spectra obtained at pH 6.08 [30]. The spectra recorded at other pH values produced weak and only a few non-sequential and sequential NOEs. The structural features observed in this work were Type-I β-turn involving residues 73–74 observed in the strong d_NN_ connectivity [30]. Hence, for Type-I β-turns a value of 2.6 Å can be expected which was observed in the d_NN_ (i + 2, i + 3) NOE connectivity [35]. Another region of the peptide, in which a specific conformation appeared, was residues 61–63, where a loop structure was observed [30]. Later, the obtained NOE constraints from this work were used in a molecular modelling study reported by the same authors [31]. It was confirmed that the turns and loops in the peptide have high a degree of flexibility and mobility, which promotes availability for interactions with calcium phosphate [31]. 

The complexation of α_s1_-CN f(59–79) with calcium, fluoride and phosphate was studied by Cross et al. [28]. The NMR spectra of the complex were recorded as in the previous study [30], predominately recording DQF-COSY, TOCSY and NOESY spectra. The sequential assignment of the spin system was performed in the fingerprint region of NOESY spectra [28]. The medium range d_γN_(i, i + 2) in the region 72–74 and medium range NOE (i, i + 2) in the region assignments of d_Nα_ 64–66 and d_Nα_ 66–68 were implicated as β-turn conformations. In addition, NMR diffusion studies were used to record the radius of the core particle of the peptide complex with calcium, fluoride and phosphate resulting in a radius of approximately 2.12 nm [29]. From this work, Cross et al. [29] concluded that the secondary structure of the peptide, with or without complexing with calcium, fluoride or phosphate, reveals a similar conformation. 

α_s1-_CN f(136–196) was studied by Alaimo et al. [77], who dissolved the peptide in buffered solution of 90% H_2_O/10% D_2_O containing 10 mM Na_2_HPO_4_ and studied for structural characterization using NMR, FTIR and CD at variable temperature (10–70 °C)_._ The NMR method selection was similar as in the previously discussed studies including DQF-COSY, TOCSY and NOESY and data acquisition using the TPPI method, as described by Marion et al. [85]. The results from Alaimo et al. [77] showed increased side chain mobility as temperature increased leading to a decreased amount of extended structures. However, at temperatures as high as 70 °C, the β-turn structures and some aromatic residues retain a stable position in the peptide chain. This was related to the involvement of Pro residues in hydrophobic turns for self-association of the peptide fragment which was referred to as a heat stable “molten globule” structural center in the α_s1-_CN [77].

#### 3.2.3. α_s2_-Casein

αs_2_-CN, due to high phosphorylation (10–13 SerP residues per molecule), is the most calcium sensitive protein [6]. However, since this casein is present only in 10% of the total caseins in milk and is comparatively difficult to isolate, only a few studies involved identification of the structural features of α_s2_-casein using NMR. In αs_2_-CN, two centers of phosphorylation have been observed, including 8–16 and 56–63. As in the previous studies on caseins, the NMR was used to study structural features of a defined region of the polypeptide chain. Huq et al. [31] published sequence specific assignments of the peptide αs_2_-CN f(2–20) isolated from the parent molecule by tryptic digestion using calcium and ethanol and further purified by fast protein liquid chromatography (FPLC) and high performance liquid chromatography (HPLC). The NMR methods used were TOCSY, NOESY and DQF-COSY, recorded at pH 6.36 and temperature of −5 °C. The amino acid spin system was assigned in the fingerprint region by sequential and non-sequential NOE [35]. The study by Huq et al. [31] for the first time provided evidence of the tendency of the phosphoserine region of the peptide to form helical structure. The strong d_NN_ and weak d_αN_ NOE connectivity observed in the NOESY spectra proved existence of the helical structure in αs_2_-CN f(2–20) [31]. 

Another peptide of αs_2_-CN, f(46–70), was studied by Cross et al. [86]. They reported an NMR study of the peptide using the same methods and procedures as in the study of Huq et al. [3] and concluded that the phosphoserine motive of the peptide (46–70) reveals a similar amide chemical shift as observed in peptides β-CN (1–25), αs_1_-CN (59–79) and αs_2_-CN (2–20). However, the addition of calcium promoted distinctly variable conformations observed in the medium-range NOE connectivity in these four peptides [86].

#### 3.2.4. κ-Casein

κ-CN is known to exist on the surface of the micelle where its hydrophilic C terminus (106–169) provides steric stabilization to the micelle [1]. Several studies that include the elucidation of the structure of peptides from the κ-CN molecule using NMR methods have been reported in the literature. Plowman et al. [33] presented complete chemical assignment of the peptide κ-CN f(98–111) in an attempt to predict the secondary structure. In this study, 20 mM of the peptide was suspended in DMSO-d_6_ and studied at pH 3.0 and 7.5 and temperatures in the range 22–58 °C. The NMR methods used were DQF-COSY, TOCSY and ROESY performed in the phase-sensitive mode with the TPPI method, as described by Marion et al. [85]. Sequential and intra residue connectivities were accomplished using the standard assignment method described by Wüthrich [35]. The study by Plowman et al. [33] revealed the existence of extended or random structure of the peptide in solution with trans isomerism of the proline residues. Moreover, a pH increase from 3.0 to 7.5 resulted in a shifting of His residues downfield as a result of the deprotonation of the side chain protons of the amino acid. The temperature changes from 22 to 58 °C resulted only in small variations in the hydrogen bonding of the NH protons of His and Leu [33].

A larger portion of the N-terminus of κ-CN, κ-CN f(1–44) was studied by Bansal et al. [34]. In this study, 1 mM of peptide was dissolved in H_2_O/D_2_O/TFE (60/10/30) or D_2_O/TFE (70/30). The standard NMR methods for structure observation were selected, including DQF-COSY for through bond coupling (^3^J_HN-αH_), TOCSY for amino acids pattern and NOESY for structure calculation using NOE distance and dihedral angle restraints. The structure calculation was based on 11 dihedral angle restraints and 375 NOE restraints including intra residue, sequential, medium and long-range. This study, for the first time, reported the presence of a defined helix between Ile_28_ and Arg_34_ and an irregular helix between Ile_9_ and Pro_27_ in the N terminal of κ-CN [34]. This was the first study that involved a structural observation of the casein peptide with a significant length using NMR. 

For studying k-CN f(130–153), Plowman et al. [16] synthetized this peptide and dissolved it in H_2_O/D_2_O (90/10, v/v) or ^2^H labelled TFE (trifluoroethanol). Similar to the previously discussed studies, the NMR methods used were DQF-COSY, TOCSY, ROESY, NOESY as the basic NMR experiment for sequential assignment of the polypeptide chain. The peptide dissolved in TFE produced a better NOE peak dispersion in the NOESY and ROESY spectra in comparison to the peptide in the aqueous solution [16]. In this study, several conformational states were observed; the amino acids from Ile_136_ to Ser_149_ existed in α-helix conformation, Thr_131_-Thr_135_ showed an unordered conformation, Glu_151_-Ile_153_ extended structures and Thr_145_-Ala_148_ a 3_10_ a helix. The Pro residues existed predominately in trans conformation [16]. 

## 4. NMR Studies on Casein Micelles

In addition to studies on specific segments on caseins, NMR has also been successfully applied to detect the position and interactions between colloidal calcium phosphate (CCP) nanoclusters and phosphoserine regions in caseins including liquid-state and solid-state ^31^P and ^43^Ca NMR [87,88,89]. The stability of CCP and its mobility was observed by Gonzalez-Jordan et al. [90]. For this purpose, they used magic-angle spinning (MAS) NMR acquiring ^1^H-^31^P cross-polarization spectra to detect the signal of immobile phosphorus. The signal for mobile phosphorus (organic and inorganic) was obtained by subtraction of the ^31^P immobile signal after integration and deconvolution. Gonzales-Jordan et al. [90] reported that 81% of organic phosphate and 97% inorganic phosphate found in the micelle are in an immobile state when milk is in its native pH (6.7). Lowering of pH to an isoelectric point leads to increased protonation of both forms of phosphate leading to precipitation of caseins. pH recovering induced reorganization of the calcium phosphate nanoclusters giving similar values of mobility as under the native conditions of milk [90]. Thus, this finding offers a crucial benefit for understanding the changes in salt equilibria in casein micelles as a result of pH cycling that is used in the dairy industry for various processes. 

Not only pH, but also temperature strongly affects the interactions among caseins and with CCP. Removal of β-casein, which is observed at low temperatures, was related to drastic changes of SerP structure and thus changes in the hydrophobicity and interaction preferences observed using liquid NMR [91]. The “loose” structure of the micelle and presence of internal passages offer advantages, such as liberation of the β-casein at low temperatures. Rollema and Brinkhuis [92] used NMR to observe casein micelle behavior as impacted by temperature (60–98 °C) and calcium removal. NMR was used in the aliphatic region of the NMR spectrum using the methyl resonances of Val, Ile and Leu, and aromatic regions for aromatic amino acids. It was observed that the caseins were characterized by great mobility, dissociated from the micelle and changed their conformational preferences at elevated temperature and calcium depletion. The changes were observed to be reversible in absence of whey proteins [92]. Combination of ^31^P NMR and FTIR can also be used to observe the influence of ionic components in milk during temperature changes [93]. Moreover, using the chemical shifts assignments of ^31^P NMR, FTIR can depict a qualitative allocation and transformation of different types of phosphorus in the milk presenting individual peaks, which are important for understanding mineral changes in casein micelles [93].

Solid-state NMR was also used to detect the presence of hydrogen bonding from non-phosphorylated parts of the caseins to amorphous calcium phosphate nanoclusters [94]. The protons of the side chains of Arg, Lys, Glu or Asp can be close in space with P atoms, with a distance of 3.4–4.4 Å, and hence the terminal groups of the side chains including R-COOH and R-NH_2_ have direct interactions with the inorganic phosphate of CCP [94]. Similarly, Cross et al. [29], observing the β-casein peptide (1–25) interactions with amorphous calcium phosphate using NMR and molecular modeling, suggested that the entire length of the peptide is involved in interactions with calcium phosphate clusters. This refers predominately to negatively charged amino acids including Glu and SerP that are not included in the phosphoserine motifs. Moreover, these interactions mainly occur in hydrophilic regions of the molecules that are responsible for post-transitional changes of caseins [29]. 

Hindmarsh and Watkinson [95] confirmed the existence of phosphorus–calcium bonding in the micelle, which is not yet classified in literature. Using ^1^H–^31^P CP-MAS NMR in Mozzarella cheese and EDTA-chelated micelles, they observed the presence of immobile phosphates complexed with calcium which are not part of the binding between phosphoserine and calcium phosphate nanoclusters. In addition, the proposed interactions included calcium linkages between individual phosphoserines in the casein micelle which can be within the same or with different caseins. However, the affinity of phosphoserines to bind calcium cation depends on their pKa which vary for individual amino acid in the polypeptide chain. The cation binding is greatest at the highest pKa. Phosphoserine centers in the caseins have a high pKa compared to individual phosphoserines in the polypeptide chain and thus are in the primary position for binding calcium ions [96]. Upon reaching saturation, other low pKa phosphoserines start to be involved in forming calcium bridges. This relationship was also confirmed using ^31^P NMR chemical shifts in caseins in the presence of calcium cations [96]. Further saturation of the casein micelles with phosphate leads to structural changes in the micelle by formation of complexes or gelation by extending or compressing of the peptide chain. This was confirmed by FTIR and ^31^P NMR on casein micelles with different phosphate content [97].

However, calcium binding to caseins balances hydrophobic interactions and electrostatic repulsions leading crosslinking and/or self-association of caseins [98]. Hydrophobic regions of caseins can form defined structures using hydrophobic interactions. Why then are they referred to as proteins with a flexible and undefined structure? The answer will likely be in the existence of high concentration of prolines in the peptide chain, which hinders the formation of defined structures and, as mentioned before, the existence of regions with hydrophilic nature. However, FTIR showed that caseins adapt certain structural features [20,32]. Hence, using FTIR and CD was confirmed for β-CN to have several defined structural regions in the molecules including helix, loops and polyproline II [20]. This was related to self-association and folding of the molecule in its hydrophobic regions. However, these methods cannot guarantee the exact position of amino acids linking. On the other hand, Malin et al. [32] included NMR in combination with FTIR, CD and molecular modeling of the N-terminal region of as_1_-CN and identified the position of individual protons of amino acids. Thus, using methods for through-bond coupling they confirmed that all prolines are in trans conformation and with through space coupling they traced sequential *d_αN_*(*i*, *i* + *j*) backbone assignments. Moreover, close positioning of amino acids leads to non-specific interactions including Van der Waals forces that allow for packing of peptide into irregular conformations [29]. 

## 5. Conclusions and Future Perspectives

NMR has been shown to be reliable and the most accurate method to observe the position of individual protons in the amino acid sequence, and thus, to identify the type of peptide connections in the protein when self-association takes place or other cross connections are established with the neighboring peptides. Thus, using multidimensional NMR methods through bond and space interactions, the complete sequential assignment and spatial orientation of a polypeptide chain can be achieved. The structure of α-LA and β-LG has been successfully observed and described using NMR and complete chemical shift assignment and tertiary structure can be found in the protein data bank. They have been fully described by multiple NMR methods, including three dimensional studies and isotopic labeling. Their globular structure and relatively short polypeptide chain have made the whey proteins of great interest to scientists as model proteins.

On the other hand, caseins, due to their conformational flexibility, have only been studied using two-dimensional NMR methods applied on specific polypeptide fragments of the molecule. Current resolved structures reported in literature using NMR proved that this method is highly reliable and can be used widely for protein studies, including caseins and casein micelle. The application of this method will enable innovative research and faster problem solutions for the industry as our understanding of the conformational behavior of these important proteins, under various relevant conditions, is unraveled.

In the dairy industry, there is a vast product evolution, generating new (and more complex) products where understanding and control of the protein structure are crucial to achieve the required structure, texture and stability. This leads to the need for using more sophisticated and powerful techniques for quality and process control. This review has demonstrated that NMR can provide knowledge of the molecular level of mechanisms in milk proteins. Since NMR has proven to be a unique tool with high sensitivity to structural changes in milk proteins, its use may become more widespread, beyond academic research. Low-resolution NMR techniques are currently applied industrially for determining, e.g., solid fat content, but with advances in the technique and improved ease of use, it may also become applicable in the future for protein characterization in dairy products and ingredients.

## Figures and Tables

**Table 1:** NMR structural studies on α-LA and β-LG, including the performed NMR methods and additional methods used. Abbreviations: DQF-COSY—double quantum-filtered correlated spectroscopy; RELAY—relayed correlation spectroscopy NOESY—nuclear Overhauser effect spectroscopy; PCIDNP—photochemically induced dynamic nuclear polarization; TOCSY—total correlated spectroscopy; ROESY—rotating frame Overhauser Effect spectroscopy; HSQC—heteronuclear single quantum coherence; CD—circular dichroism; DYANA—dynamics algorithm for NMR applications; ARIA—ambiguous restraints for iterative assignment; PCA—principal component analysis; WATERGATE—water suppression by gradient tailored excitation.

Protein	NMR Methods Used	Additional Methods	Reference
α-LA	DQF-COSY, RELAY, NOESY	PCODNP	[56]
α-LA	DQF-COSY, NOESY, RELAY		[52]
α-LA	DQF-COSY, TOCSY, NOESY, ROESY, presaturation	CD	[58]
α-LA	3D: TOCSY-HSQC, NOESY-HSQC, TOCSY, NOESY	CD	[3]
α-LA	3D: ^15^N-edited NOESY-HSQC, COSY		[53]
β-LG	DQF-COSY, TOCSY, NOESY	CD	[17]
β-LG	DQF-COSY, TOCSY, NOESY		[18]
β-LG	^13^C, ^15^N-protein labelling	CD, X-ray scattering	[59]
	2D: ^15^N-HSQC, TOCSY		
	3D: CBCA(CO)NH, HNCACB, HNCO, HN(CA)CO, HCCH-TOCSY, CCH-TOCSY, (H^β^)C^β^(C^γ^C^δ^)H^δ^ H^β^(C^β^C^γ^C^δ^)H^δ^, ^1^H-^1^H-^15^N NOESY		
β-LG	DQF-COSY, TOCSY, NOESY	Structure calculation (DYANA)	[60]
β-LG	^15^N,^13^C-labelled proteins		[61]
	2D: ^1^H-^15^N HSQC		
	3D: CBCA(CO)NH, HNCACB, HBHA(CBCACO)NH, HBHA(CBCA)NH, HNCO, HN(CA)CO, H(C)(CO)NH-TOCSY, WATERGATE		
β-LG	DQF-COSY, TOCSY, NOESY	Thermal analysis	[62]
β-LG	^13^C, ^15^N-protein labelling	Structure calculation (DYANA, X-PLOR)	[63]
	3D: ^1^H-^15^N NOESY-HSQC, ^13^C NOESY-HSQC, HNHA, HNHB		
β-LG	DQF-COSY, TOCSY, T1 and T2 relaxation studies	X-ray crystallography for modelling	[64]
β-LG	^15^N,^13^C-labelled proteins	Structure calculation (ARIA extension of X-PLOR)	[4]
	3D: ^13^C- and ^15^N-edited NOESY-HSQC, HNHA, T1 and T2 ^15^N relaxation times, WATERGATE		
β-LG	^1^H-^15^N NOESY-HSQC, ^13^C NOESY-HSQC		[65]
β-LG	TOCSY, NOESY, WATERGATE		[66]
β-LG	^15^N, ^13^C double-labelled protein	CD	[67]
	3D: CBCA(CO)NH, HNCACB, HNCO, HNCACO, relaxation analysis		
β-LG	^15^N, ^13^C double-labelled protein	PCA	[68]
	H/D Exchange experiments		
	3D: CBCA(CO)NH, HNCACB, HNCO, HNCACO		
β-LG	^1^H−^15^N HSQC; H/D exchange; transverse relaxation (R2)		[69]
	3D: HNCACB, CBCACONH, HNCO, HNCACO		

**Table 2:** NMR structural studies on peptides from caseins (β-, αs_1_-, αs_2_-, κ-casein) including the performed NMR methods and additional methods used. Abbreviations: WET - water suppression enhanced through T1 effect; sLED - suppression longitudinal encode decode. For other abbreviations see Table 1.

Protein Fragments	NMR Methods	Additional Methods	References
β-CN f(1–25)	2D: DQF-COSY, TOCSY, ROESY, NOESY		[25]
β-CN f(1–25)	2D: COSY, R-COSY, TOCSY, NOESY		[26]
β-CN f(1–25)	2D: DQF-COSY, TOCSY, NOESY, ROESY		[27]
β-CN f(1–25)	2D: DQF-COSY, TOCSY, NOESY, ROESY	Molecular modelling	[29]
α_s1_-CN f(59–79)	2D: DQF-COSY, TOCSY, NOESY, ROESY	Molecular modelling	[30,76]
α_s1_-CN f(1–23)	2D: TOCSY, NOESY, HSQC	FTIR, CD, Molecular modelling	[32]
α_s1_-CN f(59–79)	2D: DQF-COSY, TOCSY, NOESY, WET, Presaturation	sLED, X-ray scattering	[28]
α_s1_-CN f(136–196)	2D: DQF-COSY, TOCSY, NOESY, Presaturation	Far-UV CD, FTIR	[77]
α_s2_-CN f(2–20)	2D: DQF-COSY, TOCSY, NOESY, WET, Presaturation	Molecular modelling	[31]
κ-CN f(98–111)	2D: DQF-COSY, TOCSY, ROESY		[33]
κ-CN f(130–153)	2D: DQF-COSY, TOCSY, NOESY, ROESY, Presaturation	CD	[16]
κ-CN f(1–44)	2D: DQF-COSY, TOCSY, NOESY	CD, Structure calculation (X-PLOR)	[34]
